# Plasma p‐tau217, NfL, GFAP diagnostic performance and biomarker profiles in Alzheimer's disease, frontotemporal dementia, and psychiatric disorders, in a prospective unselected neuropsychiatry memory clinic

**DOI:** 10.1002/alz.70717

**Published:** 2025-09-30

**Authors:** Dhamidhu Eratne, Matthew Kang, Charles B Malpas, Christa Dang, Courtney Lewis, Oneil G Bhalala, Qiao‐Xin Li, Steven Collins, Colin L Masters, Samantha M Loi, Alexander F Santillo, Kaj Blennow, Henrik Zetterberg, Dennis Velakoulis, And The MiND Study Group

**Affiliations:** ^1^ Neuropsychiatry Centre Royal Melbourne Hospital Melbourne Victoria Australia; ^2^ Department of Psychiatry University of Melbourne Melbourne Victoria Australia; ^3^ Department of Medicine (Royal Melbourne Hospital) University of Melbourne Melbourne Victoria Australia; ^4^ Melbourne School of Psychological Sciences University of Melbourne Melbourne Victoria Australia; ^5^ National Ageing Research Institute Melbourne Victoria Australia; ^6^ Department of General Practice University of Melbourne Victoria Melbourne Victoria Australia; ^7^ Institute of Health and Wellbeing Federation University Melbourne Victoria Australia; ^8^ Department of Medicine Royal Melbourne Hospital Melbourne Victoria Australia; ^9^ Genetics and Gene Regulation Division The Walter and Eliza Hall Institute of Medical Research Melbourne Victoria Australia; ^10^ National Dementia Diagnostics Laboratory The Florey Melbourne Victoria Australia; ^11^ Clinical Memory Research Unit Department of Clinical Sciences Faculty of Medicine Lund University Lund Sweden; ^12^ Institute. of Neuroscience and Physiology University of Gothenburg Mölndal Sweden; ^13^ Clinical Neurochemistry Laboratory Sahlgrenska University Hospital Mölndal Sweden; ^14^ Neurodegenerative Disorder Research Center Division of Life Sciences and Medicine and Department of Neurology Institute on Aging and Brain Disorders University of Science and Technology of China and First Affiliated Hospital of USTC Hefei Anhui P.R. China; ^15^ Department of Psychiatry and Neurochemistry Institute of Neuroscience and Physiology the Sahlgrenska Academy at the University of Gothenburg Mölndal Sweden; ^16^ Department of Pathology and Laboratory Medicine University of Wisconsin School of Medicine and Public Health University of Wisconsin‐Madison Madison Wisconsin USA; ^17^ Wisconsin Alzheimer's Disease Research Center University of Wisconsin School of Medicine and Public Health University of Wisconsin‐Madison Madison Wisconsin USA

**Keywords:** age‐adjusted reference ranges, Alzheimer's disease, blood‐based biomarkers, diagnostic accuracy, frontotemporal dementia, GFAP, memory clinic, neurodegeneration, neurofilament light chain, plasma biomarkers, primary psychiatric disorders, prospective cohort, psychiatric misdiagnosis, p‐tau217

## Abstract

**INTRODUCTION:**

Plasma biomarkers offer promise for improving the diagnosis of Alzheimer's disease (AD) and differentiating AD and other neurodegenerative disorders (NDs) like frontotemporal dementia (FTD) from primary psychiatric disorders (PPDs), particularly in younger patients.

**METHODS:**

In this prospective study, we investigated plasma phosphorylated tau 217 (p‐tau217), neurofilament light chain (NfL), and glial fibrillary acidic protein (GFAP) in 341 unselected participants from a neuropsychiatry memory clinic, including AD (*n* = 40), behavioral variant FTD (bvFTD) (*n* = 15), PPD (*n* = 69), other NDs, and controls.

**RESULTS:**

Plasma p‐tau217 showed strong diagnostic performance for distinguishing AD from bvFTD (96% accuracy) and PPD (93% accuracy). NfL best distinguished all NDs from PPD, while GFAP did not bring additional value. Biomarker profiles using predefined cut‐offs and age‐adjusted z‐scores further clarified group differences.

**DISCUSSION:**

Plasma p‐tau217 and NfL have strong diagnostic utility in real‐world, diagnostically complex cohorts. These findings support implementation of scalable blood‐based biomarkers to improve early and accurate diagnosis in memory clinical settings.

**Highlights:**

- Plasma p‐tau217 was significantly elevated in AD compared to other disorders.
- P‐tau217 distinguished AD from bvFTD with high accuracy.
- P‐tau217 distinguished AD from PPDs with high accuracy.
- NfL/p‐tau217 ratio and GFAP added limited diagnostic value compared to p‐tau217 and NfL.
- Findings support blood biomarkers in younger, real‐world clinical cohorts.

## 1 BACKGROUND

Blood‐based biomarkers show great potential to transform diagnosis and clinical care of patients presenting with cognitive, psychiatric, and neurological symptoms. Their potential includes improving early and accurate diagnosis of Alzheimer's disease (AD) and resolving real‐world diagnostic challenges, such as distinguishing AD from other neurodegenerative disorders (NDs) like behavioral variant frontotemporal dementia (bvFTD) and from common mimics such as primary psychiatric disorders (PPDs).^1^, ^2^, ^3^, ^4^, ^5^, ^6^ Early, accurate diagnosis is increasingly important with the emergence of disease‐specific treatments, such as monoclonal antibodies targeting amyloid beta.

Two of the most promising biomarkers are plasma phosphorylated tau 217 (p‐tau217) and neurofilament light chain (NfL) protein. P‐tau217 shows strong diagnostic performance and specificity to distinguish AD from non‐AD disorders (including non‐AD dementias and PPD).^7^, ^8^, ^9^, ^10^ Within AD and control cohorts, plasma levels of p‐tau217 correlate with tau positron emission tomography (PET) pathology^11^ and tangle load at autopsy^12^ and are elevated in disorders with tangles but without amyloid plaques,^13^ supporting its reflection of tau phosphorylation and pathology. NfL, a non‐specific marker of neurodegeneration and acute neuronal injury, particularly in long myelinated axons, distinguishes neurodegenerative disorders (NDs) from ND mimics, including PPDs and non‐NDs,^3^, ^4^, ^14^, ^15^ and has utility in clinical settings.^14^, ^16^, ^17^, ^18^, ^19^ Glial fibrillary acidic protein (GFAP), a marker of astrocytic activation and neuroinflammation, is an additional biomarker of interest in a range of disorders.^14^, ^20^, ^21^, ^22^, ^23^, ^24^, ^25^, ^26^ However, the utility of GFAP for the clinical differentiation between AD, non‐AD NDs, and PPDs, especially when compared to p‐tau217 and NfL is not yet clear.

We previously published diagnostic performance, cut‐offs, and reference ranges for plasma p‐tau217 and NfL,^10^, ^14^, ^18^, ^27^, ^28^, ^29^ but we have not investigated their combined use. Furthermore, while we and others have published data showing the superiority of NfL compared to GFAP to distinguish bvFTD from PPDs,^30^, ^31^ we have not yet investigated all three, p‐tau217, NfL, and GFAP, in a younger real‐world clinical neuropsychiatric cohort with diverse NDs and PPDs. Some studies have investigated combinations of these markers. Benussi et al. investigated NfL, p‐tau217, NfL/p‐tau217 ratios, and other biomarkers in 374 participants (97 AD, 278 FTD).^32^ They found strong diagnostic performance of p‐tau217 and slightly superior performance of NfL/p‐tau217 to distinguish between AD and FTD. However, they lacked a PPD group, which is critical to appreciate the use of biomarkers for bvFTD diagnosis. Rousset et al. assessed p‐tau217 and NfL in an unselected memory clinic cohort, showing strong utility of p‐tau217 for AD diagnosis and describing biomarker profiles (high/low p‐tau217 and NfL) across clinical groups.^17^ To our knowledge, no study to date has jointly investigated p‐tau217, NfL, GFAP, and biomarker profiles, with a specific focus on AD, bvFTD, PPD, in younger people, where differential diagnoses, diagnostic uncertainty, misdiagnosis, and delay are greater.^1^, ^33^, ^34^, ^35^ Further research is needed to properly establish the roles of single and combination biomarkers in real‐world clinical settings with diverse neuropsychiatric cohorts.

The primary aim of this study was to compare levels and diagnostic performances of plasma p‐tau217, NfL, NfL/p‐tau217 ratio, and GFAP for distinguishing between AD, bvFTD, and PPDs. We hypothesized that combining p‐tau217 and NfL would improve diagnostic accuracy compared to either alone. We also aimed to describe p‐tau217/NfL biomarker profiles of AD pathology and neuronal injury (e.g., low AD/low neuronal injury, high AD/high neuronal injury) in an unselected population of patients from a neuropsychiatry memory clinic. We focused on p‐tau217 and NfL using our previously described cut‐off for plasma p‐tau217^10^ and age‐adjusted *z*‐score reference range models for plasma NfL.^27^, ^30^ We wanted to explore whether GFAP could improve any distinctions, for example, between bvFTD and PPDs. Although some studies reported age‐binned reference ranges for GFAP in serum^36^ and plasma,^37^ we are unaware of any studies that have described GFAP reference ranges using continuous modeling to derive precise age‐adjusted percentiles and *z*‐scores, as we did for NfL. As an exploratory aim, we applied a novel *z*‐score model for plasma GFAP to define biomarker profiles reflecting AD pathology, neuronal injury, and neuroinflammation (p‐tau217/NfL/GFAP).

## 2 METHODS

The current study included participants prospectively recruited between June 2019 and April 2023 who had provided a blood sample for biomarker analysis. This is a follow‐up to our previous study cohort in which we investigated plasma and cerebrospinal fluid (CSF) NfL.^14^ In the current study, we included participants who had plasma p‐tau217, NfL, and GFAP biomarker results available. In the current study the focus was on AD, bvFTD, and PPD diagnostic groups, with comparator groups being control participants and other NDs (both described in the previous study). Data were available in the current study for additional comparator groups (not previously described): people with mild cognitive impairment (MCI) and people with presymptomatic genetic NDs. The bvFTD group included patients meeting diagnostic criteria for possible bvFTD. The participants in this study did not overlap with our previous study on NfL and GFAP in bvFTD, mood, and psychotic disorders.^30^

**RESEARCH IN CONTEXT**

1. **Systematic review**: We reviewed the PubMed literature on plasma p‐tau217, NfL, and GFAP in AD, FTD, and PPDs. Although several studies support the individual diagnostic value of p‐tau217 and NfL, few examined all three biomarkers together in real‐world, diagnostically heterogeneous clinical cohorts, including PPDs.
2. **Interpretation**: This prospective study demonstrates the strong diagnostic performance of plasma p‐tau217 in distinguishing AD from FTD and PPDs and other non‐AD disorders and confirms NfL as a useful marker of neurodegeneration. GFAP added limited value. These findings add to growing evidence on the clinical application of blood‐based biomarkers in younger and diagnostically complex populations.
3. **Future directions**: Future research should validate these findings in larger, more diverse cohorts, develop multimodal diagnostic algorithms, and evaluate the clinical utility and cost‐effectiveness of implementing blood biomarker testing in psychiatric, primary care, and memory clinic settings.

Participants were recruited from the Neuropsychiatry Centre at The Royal Melbourne Hospital, a quaternary service receiving referrals for diagnostically complex cases from primary care and other specialist services within Australia. Patients, as part of routine clinical care through the Neuropsychiatry Centre, received comprehensive multidisciplinary assessments and multimodal investigations, including CSF AD biomarker analysis, with gold standard consensus diagnosis based on established diagnostic criteria, as previously described in detail.^14^, ^18^, ^28^ Diagnostic group categorization was determined based on the most recent diagnosis, at longitudinal follow‐up, based on established diagnostic criteria, blinded to plasma biomarker levels, as previously described.^14^, ^18^, ^28^ Control participants were people recruited from the community. Cognitive screening data are still being collected and not yet available for this study; however, no control participants had symptoms or diagnoses of neurological or NDs and no active psychiatric symptoms or conditions.

EDTA plasma samples were collected during patients’ diagnostic work‐up and at first visit for community controls. Samples were stored at −80°C. Plasma NfL and GFAP were measured using N2PB kits on a Quanterix Single molecule array (Simoa) HD‐X analyzer, according to the manufacturer's instructions (Quanterix Corp., Billerica, MA, USA). Plasma p‐tau217 was measured using an in‐house University of Gothenburg (UGOT) p‐tau217 assay, as previously described in detail.^38^ The measurements were performed in one round of experiments using one batch of reagents by analysts blinded to clinical data and diagnoses, thereby reducing potential batch effects.

This study, part of the Markers in Neuropsychiatric Disorders Study (The MiND Study, https://themindstudy.org), was approved by the human research ethics committee at Melbourne Health (2016.038, 2017.090, 2018.371, 2020.142).

### 2.1 Statistical analyses

Statistical analyses were performed using R version 4.5.0 (2025‐04‐11). Biomarker levels in different groups were compared using standardized bootstrapped general linear models (GLMs), with age at blood sample and sex as additional covariates. Receiver operating characteristic (ROC) curve analyses were then performed to investigate diagnostic utility between different combinations of groups. Bootstrapped differences in area under the curve (AUC) were used to compare ROC curves. Optimal cut‐offs were selected based on Youden's *J* statistic. Additional diagnostic test parameters were computed: positive and negative likelihood ratios, positive and negative predictive values, overall accuracy, and diagnostic odds ratio. Additional sensitivity analyses were performed: excluding extreme outliers, excluding patients with Creutzfeldt–Jakob disease (CJD), and performing all GLMs with weight included as a covariate. As results were similar, the results excluding weight and including outliers and patients with CJD were presented to maximize the sample sizes for analyses and presented results (since not all participants had weight data).

We focused on describing p‐tau217 and NfL biomarker profiles. These were low p‐tau217/low NfL (a low AD pathology and low neuronal injury profile – a “normal” profile) and abnormal profiles: low p‐tau217/high NfL (low AD pathology but elevated neuronal injury), high p‐tau217/low NfL (AD pathology but low neuronal injury), and high p‐tau217/high NfL (high AD pathology and high neuronal injury). Plasma p‐tau217 and NfL biomarker levels were dichotomized in to “high” and “low” based on our previously published data and cut‐offs that used the same assays.^10^, ^27^ For p‐tau217, we used a cut‐off of 2.35, which was optimal at distinguishing AD from non‐AD, as previously described and published.^10^ For plasma NfL, given the strong non‐linear association with age, we used age‐based percentiles and *z*‐scores derived from the generalized additive models for location, scale, and shape (GAMLSS) model of a large reference control cohort that we developed, previously described and published,^14^ defining the 95th percentile as the cut‐off between high and low. For this study, we created a novel age‐adjusted GFAP reference range model using (GAMLSS) using this study's control group. This allowed us to derive precise age‐based percentiles and *z*‐scores and thus more precise categorization, again given the significant strong and non‐linear association with age compared to coarse age‐binned cut‐offs. The 95th percentile was defined as the cut‐off for high and low GFAP levels.

## 3 RESULTS

The final cohort consisted of 341 participants: 40 with AD (median age 62 years, 53% female), 15 with bvFTD (median age 57 years, 27% female), and 69 with PPDs (median age 55 years, 48% female). There were 119 controls (median age 63, 74% female), and in the additional comparator groups: 67 with other NDs, 13 with MCI, and 18 with presymptomatic genetic NDs (see Table 1 for full details).

**TABLE 1:** Study cohort details and biomarker levels.

Characteristic	*N*	AD *N* = 40^a^	bvFTD *N* = 15^a^	PPD *N* = 69^a^	Control *N* = 119^a^	Other ND *N* = 67^a^	MCI *N* = 13^a^	Presymptomatic genetic ND *N* = 18^a^
Age	341	62 (58, 65)	57 (56, 62)	55 (45, 62)	63 (55, 70)	61 (45, 67)	65 (56, 67)	51 (43, 62)
Sex	341							
Female		21/40 (53%)	4/15 (27%)	33/69 (48%)	88/119 (74%)	29/67 (43%)	3/13 (23%)	13/18 (72%)
Male		19/40 (48%)	11/15 (73%)	36/69 (52%)	31/119 (26%)	38/67 (57%)	10/13 (77%)	5/18 (28%)
Weight	270	73 (59, 83)	84 (63, 101)	84 (73, 99)	75 (65, 85)	75 (65, 88)	84 (78, 90)	79 (70, 98)
Unknown		15	2	14	16	12	4	8
p‐tau217	341	3.63 (2.90, 4.41)	1.07 (0.72, 1.50)	0.92 (0.58, 1.32)	0.91 (0.65, 1.31)	1.11 (0.69, 1.68)	1.04 (0.78, 1.40)	0.83 (0.65, 1.25)
Nfl	341	24 (18, 28)	21 (12, 55)	11 (8, 13)	12 (9, 17)	29 (16, 42)	15 (13, 20)	12 (10, 19)
NfL/p‐tau217 ratio	341	7 (5, 9)	25 (10, 48)	12 (9, 20)	13 (9, 22)	24 (11, 47)	16 (12, 27)	14 (11, 27)
GFAP	341	212 (151, 305)	79 (53, 192)	86 (56, 117)	115 (89, 177)	132 (77, 216)	144 (86, 174)	97 (61, 125)
log p‐tau217	341	0.56 (0.46, 0.64)	0.03 (−0.14, 0.18)	−0.04 (−0.24, 0.12)	−0.04 (−0.19, 0.12)	0.05 (−0.16, 0.23)	0.02 (−0.11, 0.15)	−0.08 (−0.19, 0.10)
log NfL	341	1.38 (1.25, 1.45)	1.31 (1.08, 1.74)	1.02 (0.90, 1.11)	1.09 (0.94, 1.24)	1.46 (1.20, 1.62)	1.19 (1.11, 1.30)	1.06 (0.99, 1.27)
log GFAP	341	2.33 (2.18, 2.48)	1.90 (1.72, 2.28)	1.93 (1.75, 2.07)	2.06 (1.95, 2.25)	2.12 (1.88, 2.33)	2.16 (1.93, 2.24)	1.99 (1.78, 2.10)

**^Abbreviations:^ :**AD, Alzheimer's disease; bvFTD, behavioral variant frontotemporal dementia; GFAP, glial fibrillary acidic protein; MCI, mild cognitive impairment; NfL, neurofilament light chain; ND, neurodegenerative disorder; PPD, primary psychiatric disorder; p‐tau217, phosphorylated tau 217

^a^ Median (Q1, Q3); *n*/*N* (%).

PPDs consisted of major depressive disorder (MDD, *n* = 21), bipolar disorder (*n* = 6), functional neurological/cognitive disorder (*n* = 8), schizophrenia spectrum disorder (*n* = 16), bvFTD “phenocopy” syndrome (*n* = 2), and other PPDs (*n* = 16, which included anxiety, personality, obsessive‐compulsive, post‐traumatic stress, and undifferentiated psychiatric disorders).

Other NDs consisted of CJD (*n* = 2), dementia with Lewy bodies (*n* = 5), dementia not otherwise specified (*n* = 6), Huntington's disease (HD, *n* = 15), mixed AD/vascular (*n* = 3), substance‐related cognitive impairment/dementia (*n* = 2), vascular dementia (*n* = 5), and a range of other NDs (*n* = 29, which included autoimmune encephalitis, cerebral amyloid angiopathy, corticobasal syndrome, central nervous system vasculitis, Down syndrome, Fahr disease, metabolic disorders, Niemann–Pick Type C, Parkinson's disease, and cerebellar degenerative disorder). The presymptomatic genetic ND group consisted of AD (*PSEN1*, *n* = 2), genetic CJD (*n* = 6), HD (*n* = 6), CADASIL (*n* = 1), bvFTD (*C9orf72* *n* = 1 and *GRN* *n* = 2).

CSF biomarker analysis, including AD proteins CSF AB42 and p‐tau181 (Supporting Information Table 1), was conducted on 80 patients. Briefly, 27/40 (68%) of AD patients had CSF AD biomarkers. Most (18/27, 67%) had a CSF AD biomarker profile consistent with AD, as defined by an amyloid‐positive, p‐tau‐positive (A+T+) profile, categorized using established cut‐offs, as described in our previous study.^10^ Other profiles in the AD group were 8/27 with A+T− and 1/27 with A−T+. Two patients with AD had amyloid PET (but not CSF), and both were positive for amyloid plaques. Further information, including phenotype information for AD, are in the Supporting Information Tables 1 and 2. Forty‐seven percent (7/15) of bvFTD patients had CSF AD biomarkers (none with an A+T+ profile, 4/7 with A−T−, and 3/7 with A+T−). None of the patients in the other groups who had CSF AD biomarkers (20 in the PPD group, 21 in other NDs, and five MCI) had A+T+ profiles (further details in Supporting Information Tables 1 and 2).

### 3.1 Levels of plasma p‐tau217, NfL, NfL/p‐tau217 ratio, GFAP, in AD, bvFTD, and PPD

#### 3.1.1 Plasma p‐tau 217

**FIGURE 1:**
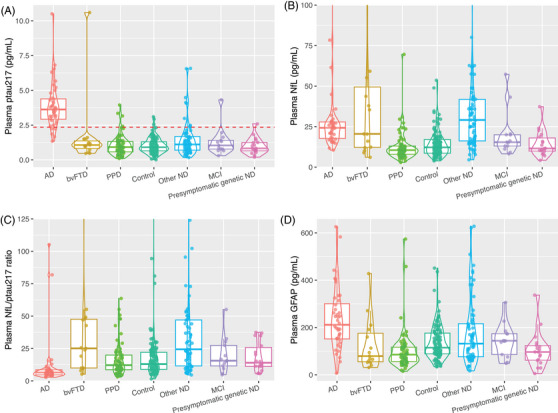
Plasma p‐tau217, NfL, NfL/p‐tau217 ratio, and GFAP levels, in AD, bvFTD, PPDs, controls, and other comparator groups. Plasma p‐tau217 levels were significantly elevated in AD compared to all other disorders and controls. To improve readability, seven outliers were not displayed in plot B for NfL (two for AD [141 and 294 pg/mL], two bvFTD [101 and 214 pg/mL], three other NDs [106, 352, and 1154 pg/mL]), and six in plot C for NfL/p‐tau217 ratio (one bvFTD [143], two controls [699, 772], three other NDs [190, 198, and 604]). Dashed red lines = predefined optimal cut‐offs from our previous studies, for p‐tau217 (2.35pg/mL). AD, Alzheimer's disease; bvFTD, behavioral variant frontotemporal dementia; GFAP, glial fibrillary acidic protein; ND, neurodegenerative disorder; NfL, neurofilament light chain; PPD, primary psychiatric disorder; p‐tau217, phosphorylated tau 217.

As demonstrated in Table 1 and Figure 1, plasma p‐tau217 levels were significantly elevated in AD compared to bvFTD (standardized bootstrapped GLM with age and sex as additional covariates; *β* = 1.30, 95% CI: [0.71, 1.73], *p* < 0.001), and AD compared to PPDs (*β* = 1.59 [1.34, 1.80], *p* < 0.001). Levels were also elevated in AD compared to the other groups (controls, Other NDs, MCI, and Presymptomatic ND, and all *p* < 0.001).

#### 3.1.2 Plasma NfL

Plasma NfL was elevated in AD compared to PPD (*β* = 0.87 [0.59, 1.13], *p* < 0.001), but not for AD compared to bvFTD (*β* = −0.10 [−0.82, 0.58], *p* = 0.788). There were no significant differences in plasma NfL levels across the ND groups (AD, bvFTD, Other NDs, all *p* > 0.05). Levels were also similar between PPDs, control, MCI, and presymptomatic ND groups.

#### 3.1.3 Plasma NfL/p‐tau217 ratio

The plasma NfL/p‐tau217 ratio was reduced in AD compared to bvFTD (*β* = −1.57 [−2.16, −0.94], *p* < 0.001) and PPD (*β* = −0.99 [−0.58, −1.34], *p* < 0.001). The ratio was also lower in AD compared to all the other groups (controls, Other NDs, MCI, Presymptomatic NDs, and all *p* < 0.002).

#### 3.1.4 Plasma GFAP

GFAP levels were elevated in AD compared to bvFTD (*β* = 0.80 [0.23, 1.39], *p* = 0.010), and PPD (*β* = 0.82 [0.36, 1.21], *p* < 0.001). GFAP levels were also elevated in AD compared to some other groups (controls, MCI, Presymptomatic ND, all *p* < 0.038), but not between AD and other NDs (*p* = 0.108).

### 3.2 Biomarker diagnostic performance to distinguish AD from PPDs, AD from bvFTD, and bvFTD from PPD

#### 3.2.1 Distinguishing AD from PPDs

**FIGURE 2:**
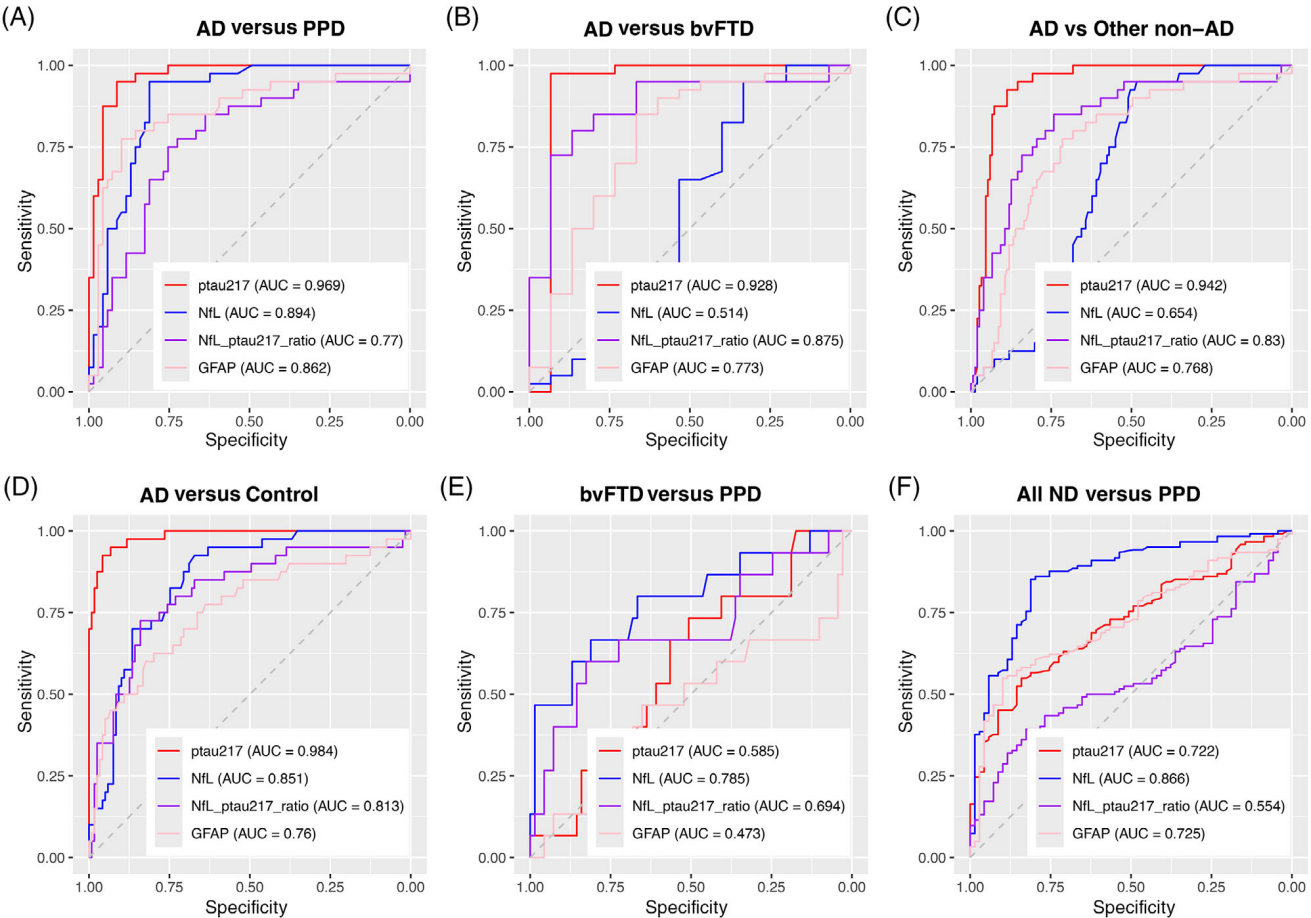
ROC analyses for diagnostic performance of plasma p‐tau217, NfL, NfL/p‐tau217 ratio, and GFAP. Plasma p‐tau217 had the very strong diagnostic performance for AD versus PPD, AD versus bvFTD, AD versus non‐AD, AD versus controls, outperforming other biomarkers and the NfL/p‐tau217 ratio. NfL's diagnostic performance was for bvFTD versus PPD and all NDs versus PPDs. AD, Alzheimer's disease; bvFTD, behavioral variant frontotemporal dementia; GFAP, glial fibrillary acidic protein; NfL, neurofilament light chain; PPD, primary psychiatric disorder; p‐tau217, phosphorylated tau 217; ROC, receiver operating characteristic.

P‐tau217 demonstrated the strongest diagnostic performance to distinguish AD from PPDs, as demonstrated in Figure 2 and Supporting Information Table 3. P‐tau217 had an AUC of 0.97, outperforming NfL, NfL/p‐tau217 ratio, and GFAP (AUCs 0.89, 0.77, 0.86 respectively; AUC differences all *p* < 0.016). P‐tau217 had the highest accuracy (93%), specificity (91%), and sensitivity (95%), at an optimal cut‐off of 1.84pg/mL. Further details of all diagnostic performance metrics are available in Supporting Information Table 3.

#### 3.2.2 Distinguishing AD from bvFTD

To distinguish AD from bvFTD, p‐tau217 once again demonstrated the highest AUC (0.93) and the strongest diagnostic performance (93% specificity, 98% sensitivity, 96% accuracy, and cut‐off 1.64) compared to the other biomarkers. NfL did not have significant diagnostic performance to distinguish AD from bvFTD (AUC 0.51 [0.30, 0.74]). The AUC difference was not statistically different between p‐tau217 and the NfL/p‐tau217 ratio (AUC 0.88, AUC difference *p *= 0.509) or GFAP (AUC 0.77, AUC difference *p* = 0.10). However, compared to p‐tau217, both the ratio and GFAP demonstrated poorer specificity (87% and 67%, respectively), sensitivity (80% and 85%), and accuracy (82% and 80%) and much lower diagnostic odds ratios (26 and 11.33, vs p‐tau217's 546).

#### 3.2.3 Distinguishing AD from non‐AD disorders

P‐tau217 demonstrated high diagnostic performance to distinguish AD from other non‐AD disorders (consisting of bvFTD, PPDs, Other NDs), with an AUC of 0.94. It was superior to NfL, NfL/p‐tau217 (all *p* ≤ 0.001) and had 88% specificity, 93% sensitivity, 90% accuracy, and a cut‐off of 2.19.

P‐tau217 demonstrated very strong diagnostic performance to distinguish AD from controls (AUC 0.98, 96% specificity, and 93% sensitivity).

#### 3.2.4 Distinguishing bvFTD from PPD

To distinguish bvFTD from PPD, NfL had the highest AUC (0.78) and strongest diagnostic performance (81% specificity, 67% sensitivity, 79% accuracy, and cut‐off 15.15pg/mL), although the AUC difference between NfL and NfL/p‐tau217 ratio was not significant (*p* = 0.14). P‐tau217 and GFAP had no diagnostic utility (AUC confidence intervals both crossed 0.50).

### 3.3 Biomarker diagnostic performance to distinguish all NDs from PPDs

As a follow‐on from our previous studies that focused on NfL in distinguishing NDs from PPDs, we investigated the ability of p‐tau217 and GFAP to distinguish NDs as a group from PPDs. We created an “all NDs” group by combining AD, bvFTD, and Other NDs. NfL demonstrated the highest AUC (0.87) and strongest diagnostic performance (81% specificity, 85% sensitivity, 84% accuracy, and cut‐off 14.35) to distinguish All NDs from PPDs, significantly outperforming GFAP (AUC 0.73, AUC difference *p* < 0.0001) and p‐tau217 (AUC 0.72, AUC difference *p* < 0.001). The NfL/p‐tau217 ratio did not have diagnostic utility for All NDs versus PPDs (AUC confidence interval crossed 0.50).xxx

### 3.4 Biomarker profiles in diagnostic groups

#### 3.4.1 AD pathology/neuronal injury (P‐tau217/NfL) biomarker profiles

We described p‐tau217 and NfL biomarker profiles in the diagnostic groups, using our previously described cutoff for plasma p‐tau217, and age‐adjusted percentiles model for NfL.^10^, ^27^

**FIGURE 3:**
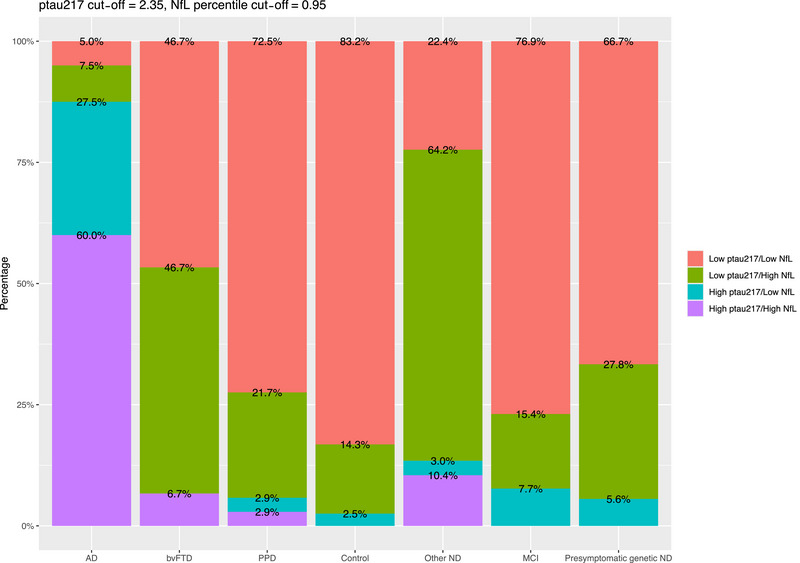
P‐tau217 and NfL biomarker profiles, classified based on previously described cut‐offs, in different diagnostic groups. NfL, neurofilament light chain; p‐tau217, phosphorylated tau 217.

As demonstrated in Figure 3, the high p‐tau217 profiles were most commonly seen in AD (total 88% of AD, 60% high p‐tau217/high NfL, and 28% high p‐tau217/low NfL biomarker profiles). By comparison less than 10% of each of the other groups exhibited high p‐tau217 profile. bvFTD only had 7% with high p‐tau217 profiles (all high p‐tau217/high NfL), PPD only 6% (3% high p‐tau217/high NfL, 3% high p‐tau217/low NfL), controls only 3% (all high p‐tau217/low NfL), and MCI and presymptomatic ND 8% and 6% respectively. Other NDs had 13% with high p‐tau217 profiles (10% high p‐tau217/high NfL, 3% high p‐tau217/low NfL).

68% of AD had high NfL profiles (high p‐tau217/high NfL, low p‐tau217/high NfL), and AD had the lowest proportion of “normal” (low p‐tau217/low NfL) profiles. For bvFTD on the other hand, only 53% had high NfL profiles, and the remaining 47% had low p‐tau217/low NfL profiles. Most PPD had a normal profile (72%), with 25% showing high NfL profiles. Looking at individual PPD (details in Supporting Information Figures 1 and 2), there was a greater proportion of patients with high NfL profiles in schizophrenia (37%) and Other PPD (33%), compared to other groups (MDD 19%, FND 12.5%, BPAD 0%), although these exploratory findings should be interpreted with caution given the small sub‐group numbers. 83% of controls had a normal profile, and only 14% having high NfL profiles. Other ND had the highest proportions of high NfL levels (74%, 64% low p‐tau217/high NfL, and 10% high p‐tau217/high NfL). Most participants in MCI, and Presymptomatic ND had low p‐tau217/low NfL profiles (72%, 77%, and 67%, respectively), similar to PPD and controls.

**FIGURE 4:**
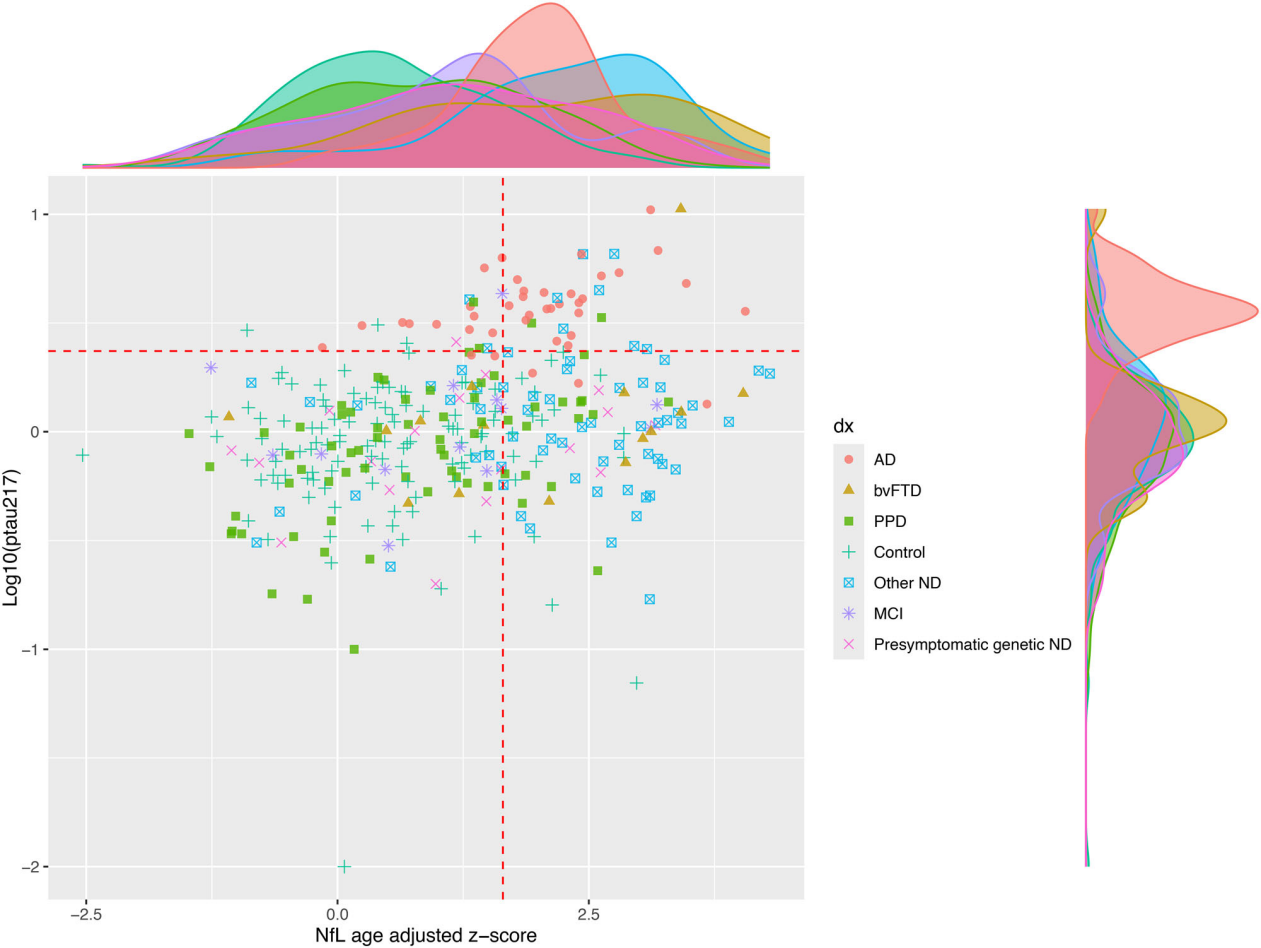
P‐tau217 and NfL levels and distributions in study cohort. The scatter plot and distributions demonstrate that almost all AD patients had elevated p‐tau217 levels and high p‐tau217/high NfL profiles, and compared to non‐AD disorders where almost all had low p‐tau217 levels. Most ND had high NfL levels, compared to a small proportion of PPD and controls. Dashed red lines = predefined optimal cut‐offs from our previous studies, for p‐tau217 (log10 of 2.35pg/mL) and age‐adjusted NfL (*z*‐score of 1.645 or 95th percentile). AD, Alzheimer's disease; NfL, neurofilament light chain; p‐tau217, phosphorylated tau 217; PPD, primary psychiatric disorder.

These findings are further demonstrated in Figure 4, a plot for different diagnostic groups of log p‐tau217 versus NfL age‐adjusted z‐score, and distributions. The scatter plot and distributions demonstrate that almost all AD patients had elevated p‐tau217 levels and high p‐tau217/high NfL profiles (most in upper right in scatter plot), as compared to non‐AD disorders, where almost all had low p‐tau217 levels. Most NDs had high NfL levels (most in lower right in scatter plot), compared to a small proportion of PPDs and controls (most in lower left in scatter plot).

#### 3.4.2 AD pathology/neuronal injury/neuroinflammation (p‐tau217/NfL/GFAP) biomarker profiles

**FIGURE 5:**
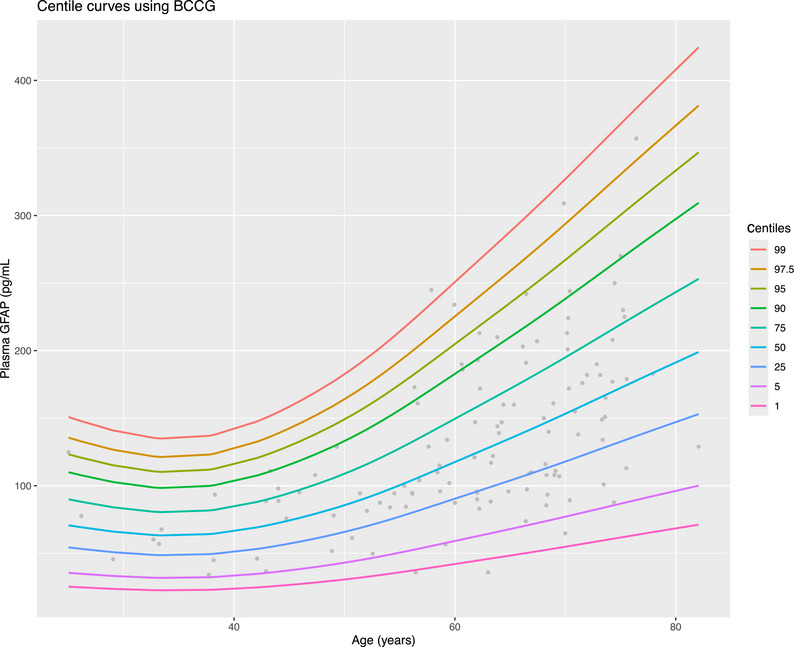
Plasma GFAP percentiles derived from generalized additive models for location, scale, and shape, based on this study's control group. Two extreme outliers were excluded for modeling (GFAP levels > 400 pg/mL, aged 52 and 63), given the disproportionate influence they would have with the relatively small sample size. GFAP, glial fibrillary acidic protein.

**FIGURE 6:**
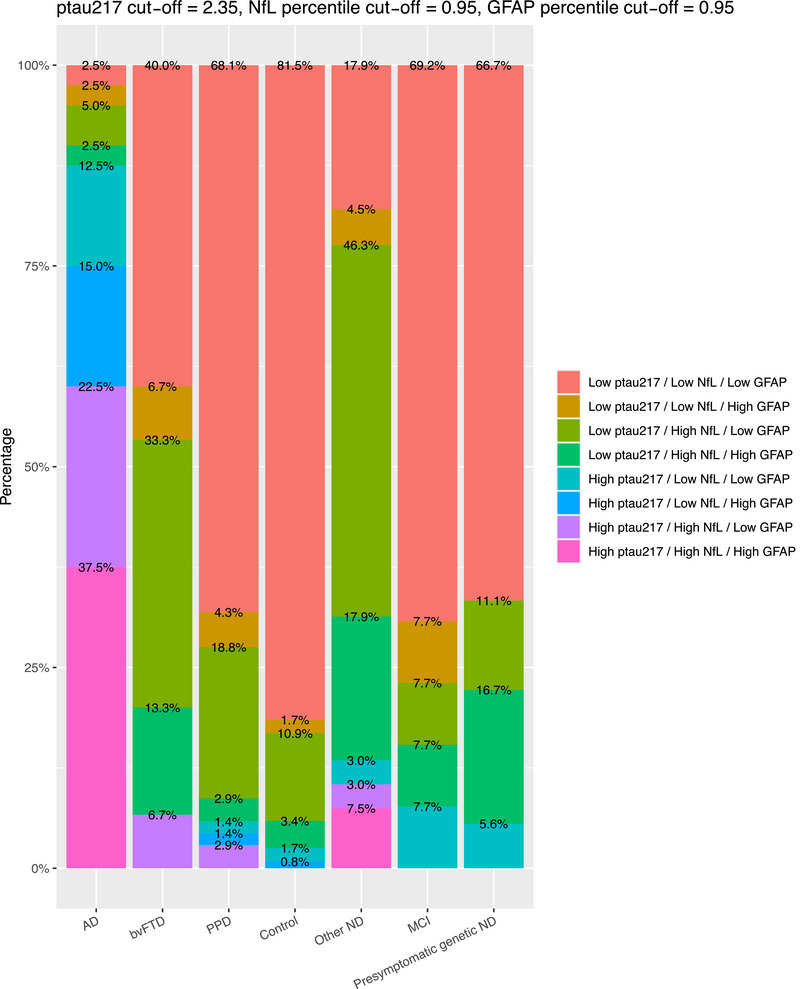
P‐tau217, NfL, and GFAP biomarker profiles in diagnostic groups. GFAP, glial fibrillary acidic protein; NfL, neurofilament light chain; p‐tau217, phosphorylated tau 217.

As an exploratory aim, we developed a novel GAMLSS age‐based model for plasma GFAP to derive percentiles and *z*‐scores, based on our control group. As demonstrated in Figure 5, this shows a similar non‐linear association with age and a U‐shaped pattern similar to the found in other studies.^36^, ^37^ This enabled complex p‐tau217/NfL/GFAP (AD pathology/neuronal injury/neuroinflammation) biomarker profiling, which is demonstrated in Figure 6. Inclusion of GFAP to create this three‐biomarker profile resulted in some differences compared to p‐tau217/NfL only profiling. The proportion of “normal” profiles using three biomarkers (i.e., low p‐tau217/low NfL/low GFAP) was slightly lower in bvFTD (40%, compared to 47% low p‐tau217/low NfL profiles). The proportion of normal p‐tau217/NfL/GFAP profiles in PPDs and controls were similar to proportions of normal p‐tau217/NfL profiles. Looking at individual PPDs (details in Supporting Information Figures 1 and 2), high GFAP profiles did not appear to be different between groups, although these findings should once again be interpreted with caution given the small subgroup numbers.

We performed some additional exploratory analyses. To investigate whether there were differences in levels of severity and biomarkers between AD and bvFTD, we first compared MMSE scores between these two groups (calculated from total scores on the Neuropsychiatry Unit Cognitive Assessment Tool (NUCOG), the cognitive screening instrument used in the Neuropsychiatry Centre).^39^ MMSE scores were not statistically different (median [interquartile range, IQR]: 21.9 [16.5 to 24.9] for AD vs 23.4 [20.9 to 25.0] for bvFTD, *p* = 0.093), Supporting Information Table 1. Next, we ran GLMs exploring whether diagnosis of AD or bvFTD, MMSE, and diagnosis × MMSE interaction influenced NfL levels, adjusting for age and sex. Diagnosis (*p* = 0.394), MMSE (*p* = 0.700), and the interaction term (*p* = 0.634) were all non‐significant. Taken together, these findings indicate that the higher proportion of “high NfL” profiles in AD was unlikely to be explained by more advanced cognitive impairment compared to bvFTD. Finally, we investigated for any associations between biomarkers and cognitive impairment in AD. In AD cases only, higher plasma NfL showed a trend toward association with lower MMSE, in GLMs also adjusting for age and sex, but it was not statistically significant (*p* = 0.076). No significant associations were observed in AD between MMSE scores and plasma p‐tau217 (*p* = 0.928) and GFAP (*p* = 0.614). These suggest that within our AD group, biomarker levels did not significantly vary with the degree of cognitive impairment.

## 4 DISCUSSION

We investigated multiple biomarkers in an unselected real‐world clinical cohort of younger participants seen in a neuropsychiatry memory clinic, focusing on AD, bvFTD, and PPDs. The main findings were as follows: (1) very strong diagnostic performance of plasma p‐tau217 to distinguish AD from bvFTD, and AD from PPDs; (2) superior performance of p‐tau217 for AD diagnosis on its own, compared to NfL/p‐tau217 ratio, NfL, and GFAP; (3) strongest performance of NfL for distinguishing bvFTD from PPDs, and all NDs from PPDs, consistent with previous studies.^30^, ^31^ This adds important further evidence to the limited literature so far on roles for different biomarkers in a real‐world clinical cohort, with p‐tau217 being a very sensitive and specific test for AD and NfL having the strongest diagnostic performance to distinguish neurodegeneration from PPDs/non‐NDs, with little role for GFAP in any of these distinctions. The key strengths of this study included a real‐world, unselected diverse, clinical, and younger cohort – a relatively underinvestigated group. Establishing the role for these biomarkers in younger people, who face greater rates of misdiagnosis and diagnostic uncertainty, is important for increasing clinical and research access, where arguably the clinical, treatment, and broader psychosocial benefits could be greater.

This study builds on our previous work on p‐tau217,^10^ providing further evidence of very strong diagnostic performance for some of the most common diagnostic distinctions, distinguishing AD from bvFTD, and AD from PPDs. We saw very high specificity and sensitivity (91% and 95%), establishing a role for such an accurate test (93%) in younger people. We found no benefit of NfL/p‐tau217 ratio over p‐tau217 alone in terms of improving the distinction between AD and bvFTD, and bvFTD from PPD, in contrast to Benussi et al.^32^ This difference in their study was slight and may reflect their older cohort and greater FTD sample sizes. Although there has been interest in GFAP assisting with AD versus non‐AD and ND versus PPD/non‐ND distinctions, we found little to no utility for GFAP. Importantly, while different p‐tau217 assays correlate well with each other, they have not been standardized with each other and thus differ in absolute concentrations.^9^ Assay standardization projects are under way; however, at present cut points must be regarded as assay‐specific and, on the very safe side, as lab‐specific.

An additional strength of the study was defining and describing biomarker profiles using previously published cut‐offs and reference ranges.^10^, ^27^, ^40^ Although the previous p‐tau217 study and cut‐off had some overlap with this study cohort, a new separate analysis of AD versus controls in this study yielded a very similar cut‐off of (2.18 pg/mL, Supporting Information Table 3). The NfL reference range was from a completely independent, large reference cohort.^27^, ^40^ We found higher p‐tau217 diagnostic performance (AUC 0.97 for AD vs PPD, and 0.98 for AD vs controls, sensitivities and specificities, and accuracies > 90%), compared to Rousset et al. (AUCs approximately 0.78 to 0.81 depending on the group being compared to AD),^17^ and we also found greater proportions of high p‐tau217/high NfL and high p‐tau217/low NfL profiles in our AD group. Our findings may reflect our younger AD group (median 62 years of age vs 66). Although most patients with PPDs had low p‐tau217/low NfL profiles, there were still a number with other profiles, including 25% with high NfL. This builds on numerous other studies that demonstrated that, although very elevated NfL levels are rarely seen in PPDs, slightly elevated levels can be seen in numerous PPDs, and PPDs cannot be assumed to be equivalent to controls/non‐neurological controls.^3^, ^4^, ^25^, ^26^, ^27^, ^30^, ^41^ Although some interesting patterns could be observed between individual PPDs (Supporting Information Figures 1 and 2), any confident interpretations are limited by the small sample sizes, and deeper investigations in larger cohorts are warranted.

Among AD, bvFTD, and other ND, we found the greatest proportion (47%) of “normal” AD/neuronal injury biomarker profile (Low p‐tau217/Low NfL) in bvFTD. This was reduced slightly when considering “normal” AD/neuronal injury/neuroinflammation (low p‐tau217/low NfL/low GFAP) profile (40%). This could reflect issues previously described with NfL technical/analytical/reference range issues^10^, ^42^ or, more likely, highlights greater complexity and clinical and biomarker heterogeneity, seen in bvFTD.^43^ Although p‐tau217 can accurately distinguish frontal AD from bvFTD, and NfL has strong diagnostic performance for bvFTD versus PPDs at group levels, our findings suggest that many individuals with bvFTD would still have non‐diagnostic biomarker levels and profiles. We found weaker diagnostic performance of NfL for bvFTD versus PPD distinction in this study compared to one of our previous studies that compared bvFTD to PPD^27^ and other studies.^41^ Patients in this study were from within the same unselected clinical cohort, whereas those other studies had patients from separate clinics/cohorts. Therefore, differences in diagnostic performance most likely reflect differences in cohorts, with the present study reflecting the performance, and limitations and ongoing challenges, in a real‐world, unselected clinical cohort. Studies are needed and under way to more deeply understand this discordance in larger groups of people with bvFTD in real‐world clinical cohorts, including multimodal neuroimaging, clinical, multi‐omic, and genomic data, to ultimately improve upon single diagnostic biomarker performance for more accurate diagnosis of bvFTD. Describing biomarker profiles were secondary, exploratory aims of this study. A limitation was binary/dichotomous “low”/“high” classification. Binary classification loses important nuance in interpreting an individual level (e.g., an individual with level 1% below the binary cut‐off or 95th percentile, and someone with a level 50% below the cut‐off or 95th percentile, would both be classified as “low”). Unfortunately, small sample sizes prevented more nuanced categorization (e.g., “high,” “low,” and “borderline”).

There was a very high p‐tau217 outlier in the bvFTD group. They had a complex history, initially diagnosed with AD based on memory impairment and neuroimaging. They subsequently developed significant personality and behavior change and eventually developed motor neuron disease (MND). At that point, the diagnosis was revised to bvFTD‐MND. Notably, their blood sample and biomarker level was prior to MND onset. Their elevated p‐tau217 level possibly reflected skeletal muscle source of p‐tau217,^44^ or it could suggest that they were unfortunate to have multiple pathologies, such as AD, as well as MND, and possibly FTD. Unfortunately, this patient did not have genetic testing, CSF AD protein analysis, or amyloid PET. It is possible that blood‐based biomarkers could help improve not only accurate AD diagnosis but also identification of cases of multiple co‐pathologies, especially important for older people, where co‐pathology is more common, and in this era of increasingly available disease‐specific treatments.^45^

The study's limitations include the single‐center, cross‐sectional design, relatively small FTD subgroup, and small AD variant subgroups preventing deeper analyses, a lack of gold standard reference tests for AD (CSF AD proteins and amyloid PET) for all participants, reliance on diagnostic categorization based on gold standard clinical diagnoses over biomarker profiles (and thus the range of CSF profiles, such as an A−T+ CSF profile for one patient with an AD diagnosis, although their CSF AB42 was borderline), and lack of genetic or *post mortem* diagnostic confirmation. Additional exploratory analyses indicated that cognitive severity did not differ significantly between AD and bvFTD and was not significantly associated with plasma biomarkers within AD, suggesting that observed biomarker patterns were unlikely to be driven by disease stage; however, these findings need to be interpreted with caution given the small sample sizes. Other NDs, MCI, and presymptomatic genetic NDs were included as comparator groups for secondary/exploratory/descriptive analyses, but their small sample sizes limited further in‐depth analyses or interpretations. While all cases had systematic longitudinal diagnostic data collection, it was beyond the scope of this study to systematically review all cases with discordant biomarkers (e.g., high NfL in PPD), for reasons of discordance and potential misclassification. Such detailed investigations will be required in future studies. Our control group relied on self‐reports since cognitive screening data on controls were not available for this study. While we could not completely rule out the possibility of some control participants with MCI due to this significant limitation, the relatively younger age, narrow range, and mainly normal biomarker levels in this group suggest that this is very unlikely to be a significant issue. We developed a novel GAMLSS plasma GFAP model. While we found a U‐shaped curve (higher levels in younger people, lower in midlife, then increasing again with age), similar to other studies,^36^, ^37^ our model was based on a relatively small number of individuals and therefore should be interpreted with caution. Studies are under way aiming to replicate this study's findings and to investigate the incremental validity of multiple biomarkers in a larger sample size, including imaging and other multi‐omic data, and in different clinical settings.

This study adds important evidence for the very strong diagnostic performance of p‐tau217 to distinguish AD from bvFTD and PPDs and identified important future research needs to improve bvFTD diagnosis. The strength and simplicity of single blood‐based biomarkers for AD diagnosis are particularly important in the era of increasing availability of anti‐amyloid and other disease‐specific treatments for AD. Ongoing further research will further establish the role of p‐tau217 and NfL in precision diagnostic algorithms to reduce misdiagnosis and diagnostic delay and improve clinical and research outcomes.

## CONFLICT OF INTEREST STATEMENT

The authors declare no conflicts of interest. Author disclosures are available in the supporting information.

## DECLARATION OF INTERESTS AND FINANCIAL DISCLOSURES

KB has served as a consultant and at advisory boards for Abbvie, AC Immune, ALZPath, AriBio, Beckman‐Coulter, BioArctic, Biogen, Eisai, Lilly, Moleac Pte. Ltd, Neurimmune, Novartis, Ono Pharma, Prothena, Quanterix, Roche Diagnostics, Sunbird Bio, Sanofi, and Siemens Healthineers; has served at data monitoring committees for Julius Clinical and Novartis; has given lectures, produced educational materials and participated in educational programs for AC Immune, Biogen, Celdara Medical, Eisai, and Roche Diagnostics; and is a co‐founder of Brain Biomarker Solutions in Gothenburg AB (BBS), which is a part of the GU Ventures Incubator Program, outside the work presented in this paper. HZ has served on scientific advisory boards and/or as a consultant for Abbvie, Acumen, Alector, Alzinova, ALZpath, Amylyx, Annexon, Apellis, Artery Therapeutics, AZTherapies, Cognito Therapeutics, CogRx, Denali, Eisai, Enigma, LabCorp, Merck Sharp & Dohme, Merry Life, Nervgen, Novo Nordisk, Optoceutics, Passage Bio, Pinteon Therapeutics, Prothena, Quanterix, Red Abbey Labs, reMYND, Roche, Samumed, ScandiBio Therapeutics AB, Siemens Healthineers, Triplet Therapeutics, and Wave; has given lectures sponsored by Alzecure, BioArctic, Biogen, Cellectricon, Fujirebio, LabCorp, Lilly, Novo Nordisk, Oy Medix Biochemica AB, Roche, and WebMD; is a co‐founder of Brain Biomarker Solutions in Gothenburg AB (BBS), which is a part of the GU Ventures Incubator Program; and is a shareholder of MicThera (outside submitted work). The other authors have nothing to disclose.

## CONSENT STATEMENT

All human subjects provided informed consent.

## Supporting information



Supporting Information

Supporting Information
