# Supplementary material for: Plasma p‐tau217, NfL, GFAP diagnostic performance and biomarker profiles in Alzheimer's disease, frontotemporal dementia, and psychiatric disorders, in a prospective unselected neuropsychiatry memory clinic
Source: Alzheimers Dement. 2025 Sep 30;21(10):e70717. doi: 10.1002/alz.70717 (PMC12481210; doi:10.1002/alz.70717)
Supplement: Supplementary file 2 — Supporting Information [file ALZ-21-e70717-s001.pdf]

SUPPLEMENTARY MATERIAL

Characteristic	N	AD N = 40[1]	bvFTD N = 15[1]	PPD N = 69[1]	Control N = 119[1]	Other ND N = 67[1]	MCI N = 13[1]	Presymptomatic genetic ND N = 19[1]
age	342	62 (58, 65)	57 (56, 62)	55 (45, 62)	63 (55, 70)	61 (45, 67)	65 (56, 67)	51 (43, 62)
sex	342							
Female		21 / 40 (53%)	4 / 15 (27%)	33 / 69 (48%)	88 / 119 (74%)	29 / 67 (43%)	3 / 13 (23%)	14 / 19 (74%)
Male		19 / 40 (48%)	11 / 15 (73%)	36 / 69 (52%)	31 / 119 (26%)	38 / 67 (57%)	10 / 13 (77%)	5 / 19 (26%)
weight	271	73 (59, 83)	84 (63, 101)	84 (73, 99)	75 (65, 85)	75 (65, 88)	84 (78, 90)	80 (70, 103)
Unknown		15	2	14	16	12	4	8
ptau217	342	3.63 (2.90, 4.41)	1.07 (0.72, 1.50)	0.92 (0.58, 1.32)	0.91 (0.65, 1.31)	1.11 (0.69, 1.68)	1.04 (0.78, 1.40)	0.84 (0.65, 1.25)
nfl	342	24 (18, 28)	21 (12, 55)	11 (8, 13)	12 (9, 17)	29 (16, 42)	15 (13, 20)	12 (8, 19)
nfl_ptau217_ratio	342	7 (5, 9)	25 (10, 48)	12 (9, 20)	13 (9, 22)	24 (11, 47)	16 (12, 27)	13 (8, 27)
ptau217_nfl	342	81 (58, 112)	25 (10, 57)	9 (5, 16)	11 (6, 19)	27 (14, 80)	22 (11, 25)	12 (5, 23)

**Characteristic**	**N**	**AD** N = 40[1]	**bvFTD** N = 15[1]	**PPD** N = 69[1]	**Control** N = 119[1]	**Other ND** N = 67[1]	**MCI** N = 13[1]	**Presymptomatic genetic ND** N = 19[1]
gfap	342	212 (151, 305)	79 (53, 192)	86 (56, 117)	115 (89, 177)	132 (77, 216)	144 (86, 174)	96 (54, 125)
mmse_converted_final^	54	21.9 (16.5, 24.9)	23.4 (20.9, 25.0)	NA (NA, NA)	NA (NA, NA)	NA (NA, NA)	NA (NA, NA)	NA (NA, NA)
Unknown		1	0	69	119	67	13	19
logptau217	342	0.56 (0.46, 0.64)	0.03 (-0.14, 0.18)	-0.04 (-0.24, 0.12)	-0.04 (-0.19, 0.12)	0.05 (-0.16, 0.23)	0.02 (-0.11, 0.15)	-0.08 (-0.19, 0.10)
lognfl	342	1.38 (1.25, 1.45)	1.31 (1.08, 1.74)	1.02 (0.90, 1.11)	1.09 (0.94, 1.24)	1.46 (1.20, 1.62)	1.19 (1.11, 1.30)	1.06 (0.91, 1.27)
loggfap	342	2.33 (2.18, 2.48)	1.90 (1.72, 2.28)	1.93 (1.75, 2.07)	2.06 (1.95, 2.25)	2.12 (1.88, 2.33)	2.16 (1.93, 2.24)	1.98 (1.74, 2.10)
csf_status	80							
A-T-		0 / 27 (0%)	4 / 7 (57%)	12 / 20 (60%)	0 / 0 (NA%)	6 / 21 (29%)	1 / 5 (20%)	0 / 0 (NA%)
A-T+		1 / 27 (3.7%)	0 / 7 (0%)	1 / 20 (5.0%)	0 / 0 (NA%)	2 / 21 (9.5%)	2 / 5 (40%)	0 / 0 (NA%)
A+T-		8 / 27 (30%)	3 / 7 (43%)	7 / 20 (35%)	0 / 0 (NA%)	13 / 21 (62%)	2 / 5 (40%)	0 / 0 (NA%)
A+T+		18 / 27 (67%)	0 / 7 (0%)	0 / 20 (0%)	0 / 0 (NA%)	0 / 21 (0%)	0 / 5 (0%)	0 / 0 (NA%)
Unknown		13	8	49	119	46	8	19

**Characteristic**	**N**	**AD** N = 40[1]	**bvFTD** N = 15[1]	**PPD** N = 69[1]	**Control** N = 119[1]	**Other ND** N = 67[1]	**MCI** N = 13[1]	**Presymptomatic genetic ND** N = 19[1]
amyloid_status^^	80							
A-		1 / 27 (3.7%)	4 / 7 (57%)	13 / 20 (65%)	0 / 0 (NA%)	8 / 21 (38%)	3 / 5 (60%)	0 / 0 (NA%)
A+		26 / 27 (96%)	3 / 7 (43%)	7 / 20 (35%)	0 / 0 (NA%)	13 / 21 (62%)	2 / 5 (40%)	0 / 0 (NA%)
Unknown		13	8	49	119	46	8	19
ad_status^^	80							
A+T+		18 / 27 (67%)	0 / 7 (0%)	0 / 20 (0%)	0 / 0 (NA%)	0 / 21 (0%)	0 / 5 (0%)	0 / 0 (NA%)
Other		9 / 27 (33%)	7 / 7 (100%)	20 / 20 (100%)	0 / 0 (NA%)	21 / 21 (100%)	5 / 5 (100%)	0 / 0 (NA%)
Unknown		13	8	49	119	46	8	19

[1]Median (Q1, Q3); n / N (%)

Supplementary Table 1. Study cohort details and biomarker levels including cognition in AD and bvFTD, and CSF AT (amyloid and p-tau) status

^: MMSE scores were calculated from total scores on the Neuropsychiatry Unit Cognitive Assessment Tool (NUCOG), the cognitive screening instrument used in Neuropsychiatry Centre (Walterfang M, Siu R, Velakoulis D. The NUCOG: Validity and reliability of a brief cognitive screening tool in neuropsychiatric patients. Australian and New Zealand Journal of Psychiatry 2006;40:995–1002. https://doi.org/10.1111/j.1440-1614.2006.01923.x.)

^^: Two patients in the AD group had amyloid PET imaging (but not CSF), and both positive for amyloid plaques

Out of 40 patients with AD in this cohort, 29 had gold standard biomarker analysis (27 had CSF analysis, and 2 had amyloid PET).

None of the AD cases in this cohort were genetic.
7 AD patients had posterior cortical atrophy, 1 frontal variant, 1 logopaenic primary progressive aphasia / language variant AD, and the remaining 31 had initial amnestic presentations.

Supplementary Table 2. Additional information on AD group

Categorisation	Difference	AUC	Cutoff	Spec	Sens	LR+	LR-	PPV	NPV	DOR	Accuracy
**AD vs PPD**											
ptau217	>NfL (p=0.016) >ratio (p<0.0001) >GFAP (p=0.010)	0.97 [0.94, 1.00]	1.84	91%	95%	10.93	0.05	86%	97%	199.5	93%
NfL	<ptau217	0.89 [0.83, 0.95]	14.55	81%	95%	5.04	0.06	75%	75%	81.8	86%
NfL/ptau217 ratio	<ptau217	0.77 [0.68, 0.87]	8.57	75%	75%	3.04	0.33	84%	84%	9.18	75%
GFAP	<ptau217	0.86 [0.78, 0.94]	148.5	90%	78%	7.64	0.25	82%	87%	30.51	85%
**AD vs bvFTD**											

Categorisation	Difference	AUC	Cutoff	Spec	Sens	LR+	LR-	PPV	NPV	DOR	Accuracy
ptau217	>NfL (p<0.001)	0.93 [0.80, 1.00]	1.64	93%	98%	14.63	0.03	98%	93%	546	96%
NfL		0.51 [0.30, 0.74]									
NfL/ptau217 ratio		0.88 [0.77, 0.98]	9.27	87%	80%	6	0.23	94%	92%	26	82%
GFAP		0.77 [0.62, 0.93]	115.5	67%	85%	2.55	0.23	87%	62%	11.33	80%
**AD vs Other non-AD**											
ptau217	>NfL (p<0.001) >ratio (p=0.001) >GFAP (p<0.001)	0.94 [0.91, 0.98]	2.19	88%	93%	8.21	0.08	69%	98%	97.21	90%
NfL		0.65 [0.58, 0.73]	14.6	48%	95%	1.84	0.10	33%	97%	17.78	58%

Categorisation	Difference	AUC	Cutoff	Spec	Sens	LR+	LR-	PPV	NPV	DOR	Accuracy
NfL/ptau217 ratio	>NfL (p=0.009)	0.83 [0.75, 0.91]	9.87	74%	85%	3.29	0.20	47%	95%	16.27	76%
GFAP	>NfL (p=0.017)	0.77 [0.69, 0.85]	148.5	72%	78%	2.72	0.31	42%	92%	8.65	73%
**bvFTD vs PPD**											
ptau217		0.59 [0.43, 0.74]									
NfL		0.78 [0.64, 0.93]	15.15	81%	67%	3.54	0.41	43%	92%	8.62	79%
NfL/ptau217 ratio		0.69 [0.52, 0.86]	23.13	83%	60%	3.45	0.48	43%	90%	7.13	79%
GFAP		0.43 [0.29, 0.66]									
**AD+bvFTD vs PPD**											
ptau217	>ratio (p<0.0001) >GFAP	0.86 [0.80, 0.93]	1.47	84%	78%	4.90	0.26	80%	83%	18.89	81%

Categorisation	Difference	AUC	Cutoff	Spec	Sens	LR+	LR-	PPV	NPV	DOR	Accuracy
	(p=0.026)										
NfL	>ratio (p=0.001) >GFAP (p=0.044)	0.86 [0.80, 0 93]	14.55	81%	87%	4.63	0.16	79%	89%	29.54	84%
NfL/ptau217 ratio	<ptau217 <NfL	0.64 [0.54, 0.75]	8.57	75%	58%	2.36	0.55	65%	69%	4.26	68%
GFAP	<ptau217 <NfL	0.77 [0.68, 0.86]	148.5	90%	65%	6.45	0.38	84%	77%	16.78	79%
**ND vs PPD**											
ptau217	<NfL	0.72 [0.65, 0.79]	1.45	84%	55%	3.44	0.54	86%	51%	6.42	65%
NfL	>ptau217 (p<0.001) >GFAP (p<0.0001)	0.87 [0.81, 0.92]	14.35	81%	85%	4.52	0.18	89%	86%	24.89	84%

Categorisation	Difference	AUC	Cutoff	Spec	Sens	LR+	LR-	PPV	NPV	DOR	Accuracy
NfL/ptau217 ratio		0.55 [0.47, 0.64]									
GFAP	<NfL	0.73 [0.65, 0.80]	148.5	90%	55%	5.41	0.50	91%	53%	10.79	68%
**Additional comparisons**											
**AD vs Controls**											
ptau217		0.98 [0.97, 1.00]	2.18	96%	93%	22.02	0.08	88%	97%	281.2	95%
NfL		0.85 [0.79, 0.91]	15.25	87%	93%	2.82	0.11	49%	96%	25.30	74%
NfL/ptau217 ratio		0.81 [0.73, 0.90]	7.81	84%	73%	4.54	0.33	60%	90%	13.88	81%
GFAP		0.76 [0.67, 0.85]	192	82%	60%	3.4	0.49	53%	86%	7	77%
**All ND vs Controls**											
ptau217		0.72 [0.65, 0.78]	1.84	93%	45%	6.71	0.59	87%	62%	11.39	69%

Categorisation	Difference	AUC	Cutoff	Spec	Sens	LR+	LR-	PPV	NPV	DOR	Accuracy
NfL		0.82 [0.77, 0.87]	20.15<br>14.7	87%<br>63%	66%<br>84%	4.88<br>2.28	0.40<br>0.25	83%<br>70%	71%<br>80%	12.26<br>9.24	76%<br>74%
NfL/ptau217 ratio		0.52 [0.45, 0.60]									
GFAP		0.59 [0.52, 0.66]	191.5	82%	41%	2.32	0.72	70%	58%	3.24	61%
**AD+bvFTD vs Controls**											
ptau217		0.87 [0.80, 0.94]	2.18	96%	69%	16.44	0.32	88%	87%	50.96	87%
NfL		0.82 [0.75, 0.89]	20.15	87%	67%	5.00	0.38	70%	85%	13.23	80%
NfL/ptau217 ratio		0.68 [0.58, 0.78]	7.96	83%	56%	3.35	0.52	61%	80%	6.39	75%
GFAP		0.66 [0.56, 0.76]	191.5	82%	51%	2.88	0.60	57%	78%	4.84	72%

Supplementary Table 3. Detailed diagnostic performance metrics for a range of diagnostic group distinctions

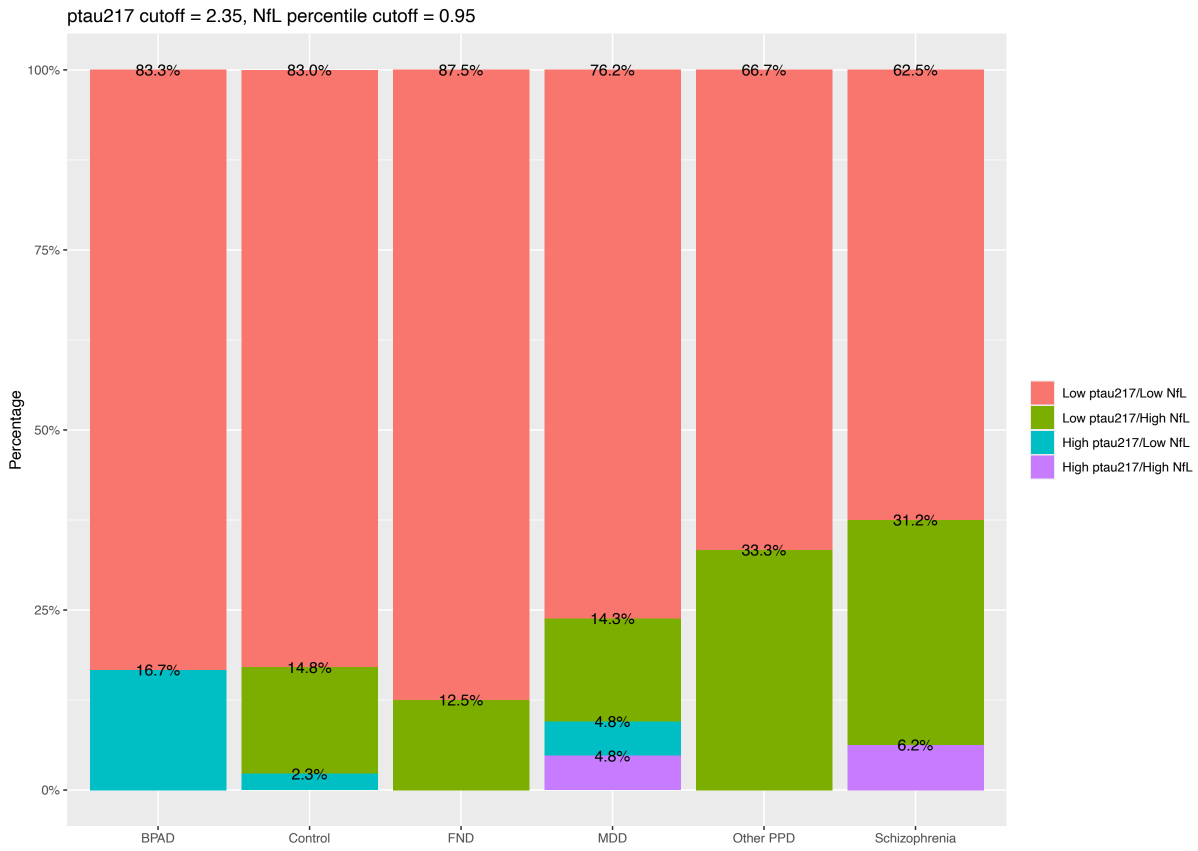

Supplementary Figure 1. Ptau217 and NfL biomarker profiles, classified based on previously described cut-offs, in different primary psychiatric disorders compared to controls.

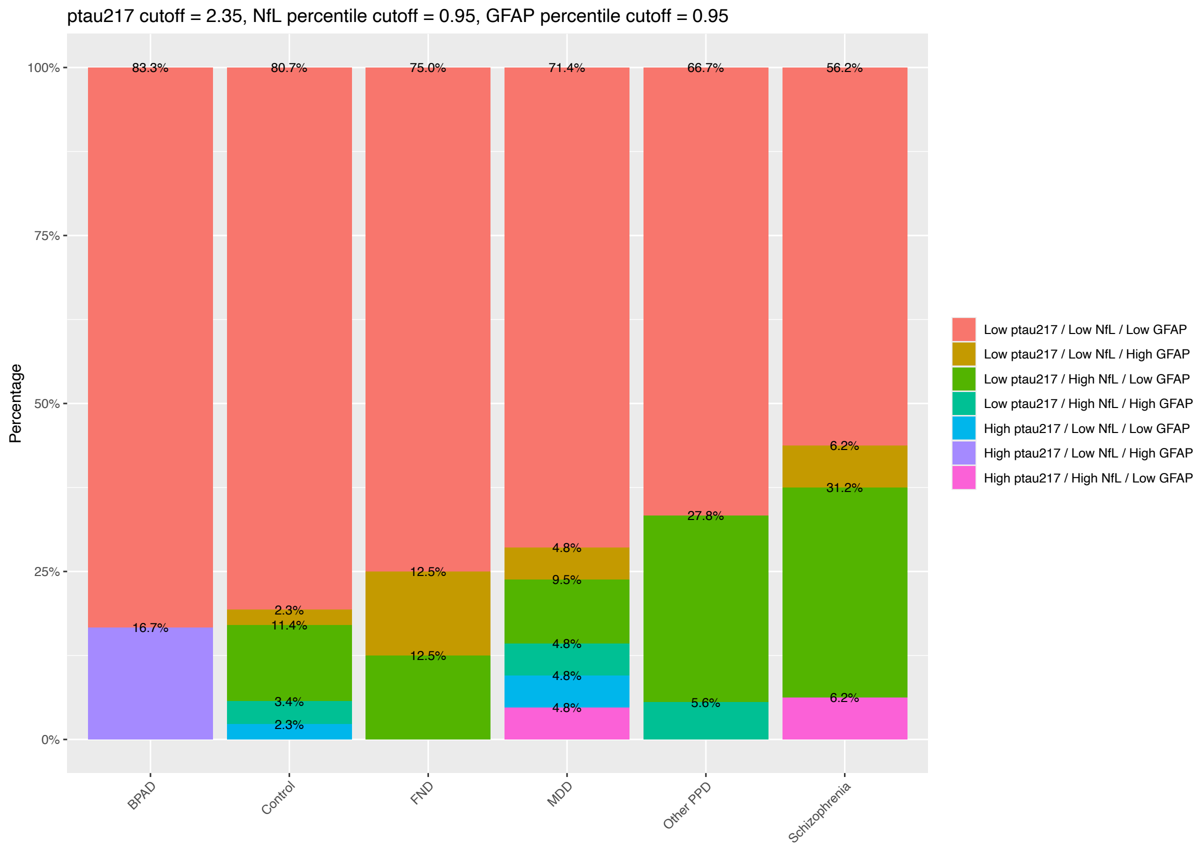

Supplementary Figure 2. Ptau217, NfL and GFAP biomarker profiles in different primary psychiatric disorders compared to controls.
